# Relationship between joint angles and gait parameters in healthy older adults aged 60 ~ 80 years

**DOI:** 10.3389/fpubh.2026.1762936

**Published:** 2026-02-13

**Authors:** Zhisheng Zhang, Xinyi Deng, Huihui Wu, Yue Liu, Tieyi Yang

**Affiliations:** 1 School of Gongli Hospital Medical Technology, University of Shanghai for Science and Technology, Shanghai, China; 2 Department of Orthopedics, Gongli Hospital of Shanghai Pudong New Area, Shanghai, China

**Keywords:** correlation analysis, gait parameters, gender differences, healthy older adults, joint angles

## Abstract

**Objective:**

This study aims to investigate the association between trunk, hip, knee, and ankle joint angles and gait parameters in healthy older adults, with a focus on analyzing gender differences, and to evaluate the independent predictive ability of joint angles for walking function.

**Methods:**

Gait analysis was performed on 60–80-year-old healthy older adults without motor dysfunction. The “Walker View” treadmill captured maximum joint angles at the trunk, hip, knee, and ankle joints, along with gait parameters during walking. Pearson correlation analysis and multiple linear regression were used to examine the relationship between joint angles and gait parameters, with separate assessments for gender differences.

**Results:**

Compared to females, males exhibited significantly greater right hip extension and bilateral ankle plantar flexion angles. Females demonstrated significantly greater bilateral knee extension and left ankle dorsiflexion angles. Trunk flexion angle negatively correlated with total steps and step frequency in males, while joint angles in females showed stronger associations with speed and step length. Stepwise regression analysis indicated that most joint angles served as independent predictors of gait parameters such as walking speed and step length, with high model fit (maximum R^2^ reaching 79%).

**Conclusion:**

Trunk and lower limb joint angles in healthy older adults are closely related to gait parameters. This suggests that gender differences should be fully considered when designing walking functional training or rehabilitation programs for older adults. Tailored exercise rehabilitation plans should be developed to delay gait function decline, enhance walking ability, and improve quality of life in older adults.

## 1 Introduction

The demographic shift toward an aging population is becoming increasingly evident, and the capacity for independent living is of paramount importance for older individuals. Gait abnormalities have been demonstrated to have a detrimental effect on the quality of life of older individuals, in addition to functioning as significant precursors to falls and disability (1). Walking speed is a widely used indicator of comprehensive gait function, and is employed in the assessment of physical fitness among older adults. Research indicates that walking speed declines with age, decreasing by approximately 12–16% every decade (2). A decline in walking speed is a significant predictor of functional decline and adverse outcomes (e.g., disability, hospitalization, and falls) in older adults (3). Consequently, the investigation of the biomechanical factors influencing walking speed and gait characteristics in older adults is of great importance.

Typical changes in gait parameters among older adults include: reduced walking speed and step length, decreased or unchanged step frequency, a more cautious and conservative gait pattern, and an increased proportion of double-support time (4). These alterations in gait are partly attributable to diminished lower-limb muscle strength and joint mobility. As individuals age, the maximum range of motion in lower-limb joints, such as the hip, knee, and ankle, gradually diminishes, while the coordination function of the joint-muscle system weakens. Research indicates that hip, knee, and especially ankle joint mobility in older adults is significantly reduced compared to younger individuals, directly contributing to a marked decrease in walking speed (5). Older adults may be compelled to adopt compensatory gait strategies in order to maintain walking stability, due to limited joint mobility. Such strategies may include increased trunk forward lean or shortened step length.

In addition to the impact of age itself, gender differences also represent a significant factor in the variation of gait characteristics observed in older adults (6). Distinct variations in body structure, muscle strength distribution, and walking habits between men and women give rise to gait parameters and joint movement patterns that are distinctly different. In general, older adults of the same age tend to exhibit larger step lengths in males, while females demonstrate relatively higher step frequencies (7). Research indicates that, even at similar walking speeds, female gait exhibits greater pelvic anterior tilt and hip flexion, along with larger knee abduction angles. Furthermore, researchers have observed that as women age, the degree of knee flexion decreases more significantly than in men, with a more pronounced reduction in knee range of motion occurring after the age of 60 (8). These gender-related disparities in gait biomechanics may signify distinct patterns in the relationship between joint angles and gait parameters among older adults of differing genders. Nevertheless, research in this area remains insufficient.

A plethora of studies have hitherto examined the disparities in mean gait metrics between older adults and younger individuals, in addition to the repercussions of pathological conditions on gait (9). Conversely, systematic research on the quantitative relationship between lower limb joint mobility and gait performance within healthy older populations remains limited. It has been hypothesized by some researchers that a lack of flexibility in the joints of the lower limbs may result in a reduction in walking speed and an alteration of the gait patterns exhibited by the subject (10). It has been posited by other researchers that an improvement in hip and ankle joint mobility can enhance both gait length and speed in older adults (11). However, the extent to which these relationships are consistent across different genders within the older population, and the relative importance of each joint in influencing gait parameters, remains inconclusive.

In consideration of the aforementioned context, the present study systematically analyses the relationship between primary joint angles and multiple gait parameters during normal gait patterns among older individuals in healthy communities. The objective of this study is to enhance comprehension of the factors that influence walking ability in China’s older population, with the ultimate aim of providing scientific substantiation for the development of interventions to enhance older adults’ gait.

## 2 Materials and methods

### 2.1 Participants

This study recruited 140 healthy older volunteers (68 males, 72 females) from the community through promotional posters. Recruitment was conducted from May 2025 to October 2025 via promotional posters and health lectures at three community health centers in Pudong New Area, Shanghai. Participants were required to be aged 60 years or older, sign an informed consent form at recruitment. After screening, they met the following inclusion criteria: (1) being in good physical and mental health without severe cardiovascular, neurological diseases, or cognitive impairment; (2) having no history of major lower limb injuries or surgeries within the past 6 months; (3) being able to walk independently for at least 5 min without assistive devices(to confirm tolerance for the 6-min gait analysis); (4) having comparable physical activity levels (engaging in moderate-intensity exercise 1–5 times/week, e.g., brisk walking, tai chi). Exclusion criteria include the presence of neurological disorders such as Alzheimer’s disease, stroke, or Parkinson’s disease. Participants with severe orthopedic conditions were excluded from the study. Furthermore, patients manifesting symptoms such as dizziness, tremors, bradykinesia, gait disturbances, postural instability, or acute pain affecting the lumbar spine, pelvis, or lower limbs during ambulation were excluded from the study. It is not obligatory for participants to fast, and all tests are conducted between 9:00 a.m. and 4:00 p.m.

### 2.2 Test program

This study employed a cross-sectional design. Each participant underwent gait testing at the Movement Function Assessment and Treatment Center of Gongli Hospital in Pudong New Area, Shanghai. All tests were conducted by the same researcher with experience in gait assessment.

### 2.3 Gait assessment

Participants underwent gait analysis using the “Walker View” treadmill (TecnoBody, Italy) at the assessment center. The apparatus has obtained ISO 13485 medical device quality management system certification, and its validity and reliability for gait analysis have been validated in clinical studies (12, 13). The system utilizes a three-dimensional motion capture system (30 fps sampling rate), comprising a well-depth infrared high-speed camera and two F-ankle sensors, to record primary joint angle data during walking. The maximum joint angles were extracted from the midpoint of the stance phase and the peak of the swing phase in the gait cycle, in accordance with the parameter extraction standards of the Walker View gait analysis module and consistent with the analytical approach of Lippi (14) in post-ICU rehabilitation research. Concurrently, the treadmill’s SCX adaptive speed control system and center-of-gravity sensing system capture gait parameters generated during the walking process. Outcome assessors were blinded to participants’ gender during data collection and analysis to reduce bias. Firstly, subjects will be required to perform a one-minute warm-up on the treadmill prior to the commencement of the formal test. The SCX system will be set to a slow walking speed of 1.0 m/s, and participants will be instructed to hold onto the handrails. This configuration enables participants to become acclimatized to the moving belt and mitigates concerns regarding balance. Once subjects have demonstrated stable gait and are able to walk without the use of a handrail, the formal 6-min walk gait analysis will be conducted. The test speed is commensurate with the subject’s adaptive pace, with the treadmill incline set at 0°. Upon completion, a bespoke report is generated, encompassing joint angle data for the trunk, hips, knees and ankles, in addition to gait parameters such as total steps, walking distance, walking speed, step frequency, step length, vertical center of mass(COM) displacement, and support time.

### 2.4 Standard protocol approval, registration, and subject consent

This study was approved by the Ethics Committee of Gongli Hospital, Pudong New Area, Shanghai (Approval No: GLYYls2025-069). The research protocol adheres to the principles of the Declaration of Helsinki and its subsequent amendments. Written informed consent was obtained from all participants.

### 2.5 Statistical analysis

**Figure 1:**
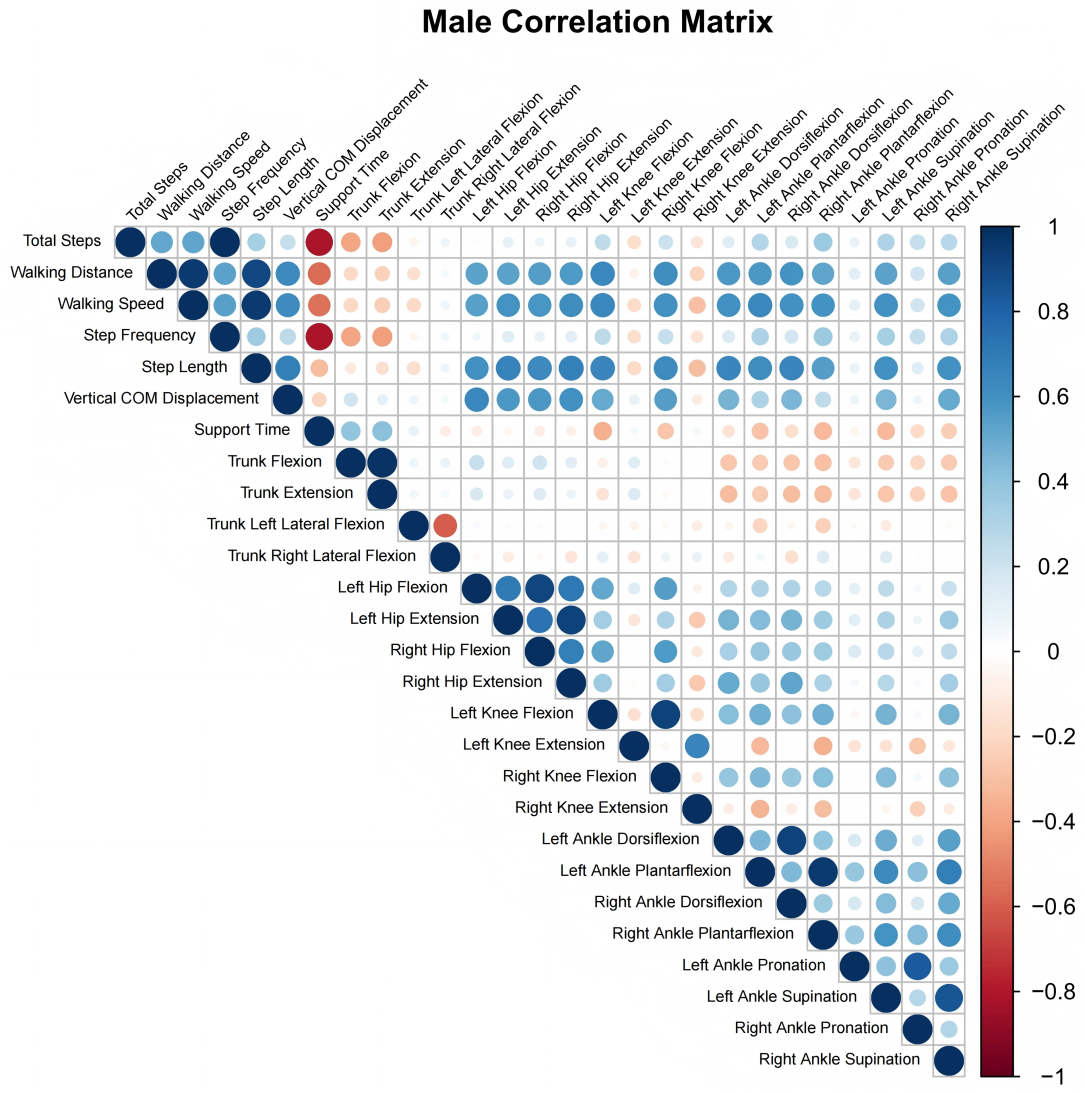
Male correlation matrix heatmap.

**Figure 2:**
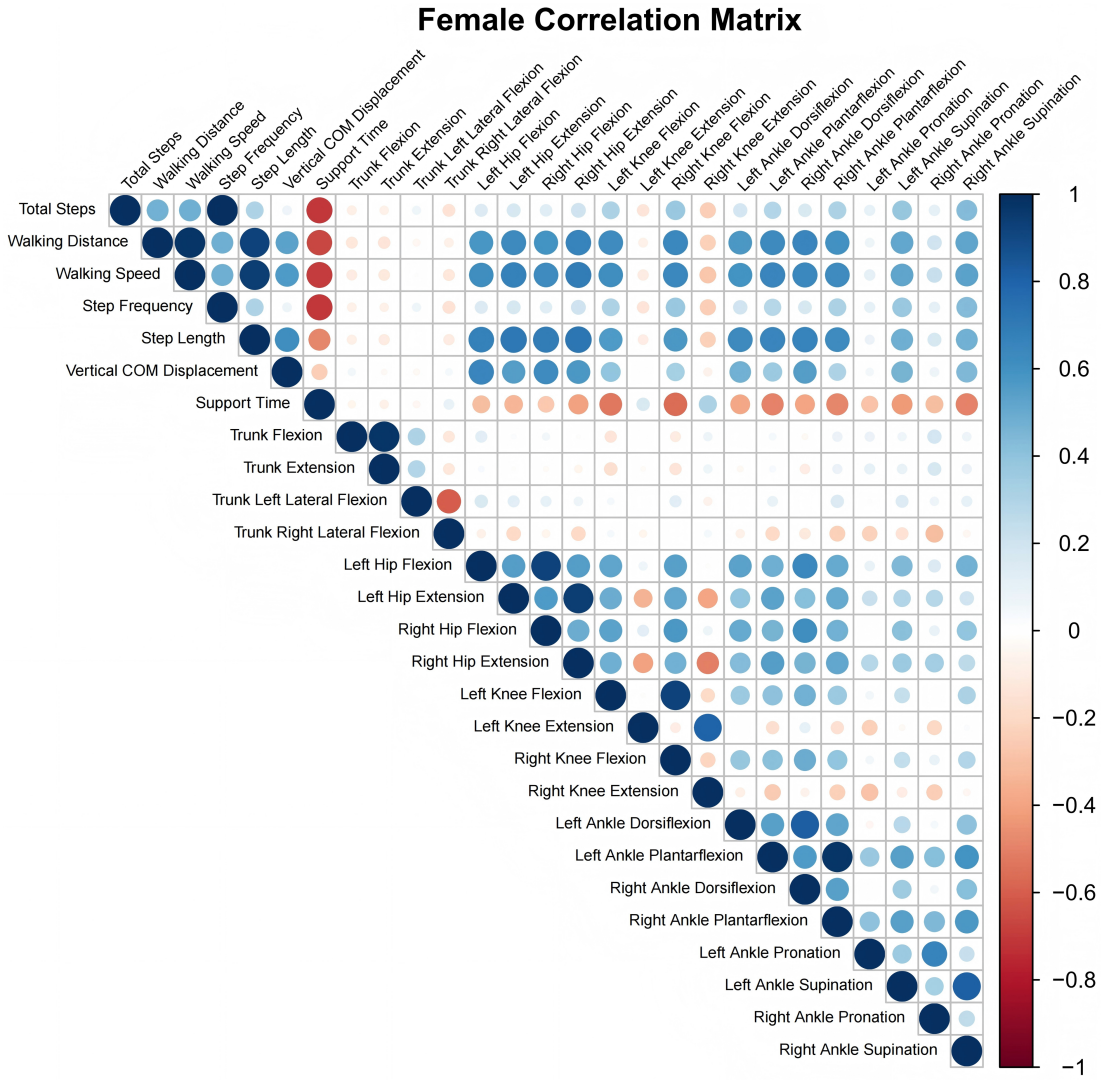
Female correlation matrix heatmap.

Initially, normality tests were conducted for all variables. Continuous variables are expressed as mean ± standard deviation. For variables that met the normality criteria, independent samples t-tests were employed; Mann–Whitney U tests were used for all other variables, with a significance level of *α* = 0.05. Pearson correlation coefficients were utilized to evaluate linear relationships between trunk, hip, knee, and ankle joint angles and various gait spatiotemporal parameters. Correlation coefficients r were calculated separately for male and female subsamples, and their significance was tested. According to Schober et al.’s (12)classification, correlatio coefficients of 0.10 ≤ r ≤ 0.39 indicate weak correlation, 0.40 ≤ r ≤ 0.69 indicate moderate correlation, and r ≥ 0.70 indicate strong correlation. Statistically significant values were defined as *p* < 0.05. In order to facilitate the presentation of results, a 3 × 7 matrix heatmap was constructed. The Pearson correlation coefficients between joint angles and seven gait parameters are represented by varying shades of color, with data displayed separately by gender (as shown in Figures 1, 2). Additionally, we employed regression models with joint angles as independent variables and seven gait parameters as dependent variables to analyze the independent predictive effects of each joint angle on gait parameters. To address the potential overfitting risk of stepwise regression, we performed 5-fold cross-validation and reported adjusted *R*^2^ to verify model stability. LASSO or ridge regression was not adopted because the current study’s variable dimension (12 joint angles, 7 gait parameters) did not reach the high-dimensional threshold that these methods are designed to address. Additionally, we employed regression models with joint angles as independent variables and seven gait parameters as dependent variables to analyze the independent predictive effects of each joint angle on gait parameters. To ensure sufficient statistical power, an *a priori* sample size calculation was performed using PASS 2021 software for multiple linear regression models: based on a moderate effect size (*f*^2^ = 0.15), *α* = 0.05, Power = 0.90, and 12 independent variables, the required minimum sample size was 128. Our actual sample of 140 participants was deemed adequate to meet statistical power requirements. To balance model simplicity and explanatory power, stepwise regression was used to select variables entering the model. The regression equation was considered significant when the overall model *p* < 0.05. The coefficient of determination R^2^ in the model indicates the explanatory power of the regression model, while the F-test assesses model significance. For each gait-related dependent variable, we recorded the significant joint angles entering the model along with their regression coefficients and *p*-values, marking significant variables with an“×” in the results table.

All statistical analyses were performed using R (version 4.4.1, R Core Team, 2020, Vienna, Austria).

## 3 Results

### 3.1 Gender comparison analysis of joint angles and gait parameters

This study included 140 participants (68 males, 72 females) with a mean age of 69.55 ± 6.40 years. There was no significant difference in age distribution between the two groups (*p* = 0.679).

With regard to the joint angles, a gender comparison analysis revealed several statistically significant differences. As demonstrated in Table 1, males demonstrated significantly greater bilateral hip extension angles in comparison to females (left hip: *p* = 0.003; right hip: *p* < 0.001). In contrast, female subjects exhibited greater bilateral knee extension angles (left knee: *p* = 0.005; right knee: *p* = 0.003). With regard to ankle joint angles, males demonstrated significantly greater plantarflexion angles (left ankle: *p* = 0.029; right ankle: *p* = 0.017), while their supination angles also exhibited a significant difference compared to those of females (left ankle: *p* = 0.030; right ankle: *p* = 0.004). It is noteworthy that no substantial gender disparities were identified in parameters such as trunk movement in all directions, hip and knee flexion, or ankle pronation.

**Table 1:** Age and joint angle of male and female subjects.

Age and joint angle	Total	*n* = 140	Male	*n* = 68	Femal	*n* = 72	*p*
	Mean	SD	Mean	SD	Mean	SD	
Characteristics							
Age (y)	69.55	6.40	69.21	6.83	69.88	5.99	0.679
Joint angles							
Trunk Flexion (°)	9.55	3.71	10.09	4.24	9.03	3.08	0.095
Trunk Extension (°)	7.43	3.57	7.81	4.06	7.06	3.01	0.217
Trunk Left Lateral Flexion (°)	1.67	1.07	1.59	1.03	1.74	1.10	0.531
Trunk Right Lateral Flexion (°)	1.50	1.03	1.46	0.91	1.53	1.15	0.990
Left Hip Flexion (°)	23.64	5.94	23.74	5.07	23.54	6.70	0.846
Left Hip Extension (°)	16.28	6.47	17.94	5.76	14.73	6.75	0.003
Right Hip Flexion (°)	24.01	5.71	23.99	5.26	24.03	6.14	0.962
Right Hip Extension (°)	16.68	6.52	18.62	5.45	14.85	6.94	<0.001
Left Knee Flexion (°)	48.10	9.49	48.35	10.53	47.87	8.46	0.596
Left Knee Extension (°)	4.27	5.61	3.80	7.09	4.71	3.70	0.005
Right Knee Flexion (°)	47.3	8.63	47.75	9.45	46.88	7.82	0.241
Right Knee Extension (°)	3.93	5.56	3.46	6.96	4.37	3.80	0.003
Left Ankle Dorsiflexion (°)	8.19	7.85	9.90	9.16	6.85	6.01	0.050
Left Ankle Plantarflexion (°)	44.93	19.82	48.58	18.80	41.48	20.26	0.029
Right Ankle Dorsiflexion (°)	8.31	7.84	9.87	9.03	6.83	6.24	0.097
Right Ankle Plantarflexion (°)	44.99	20.22	49.02	19.67	41.19	20.13	0.017
Left Ankle Inversion (°)	6.97	5.52	6.76	5.74	7.15	5.33	0.514
Left Ankle Eversion (°)	6.21	4.93	6.92	4.69	5.54	5.10	0.030
Right Ankle Inversion (°)	7.98	6.14	7.60	6.05	8.35	6.24	0.510
Right Ankle Eversion (°)	6.71	5.24	7.74	4.89	5.74	5.41	0.004

As demonstrated in Table 2, there is a clear indication of systematic gender differences in gait parameters. The male participants exhibited superior walking performance, as evidenced by increased walking distance (*p* = 0.013), faster walking speed (*p* = 0.005), and greater step length (*p* = 0.001). Furthermore, a statistically significant difference was observed in the vertical COM displacement exhibited by males during walking (*p* < 0.001). However, no significant differences were observed between the groups for parameters including total steps, step frequency, and support time.

**Table 2:** Gait parameters of male and female subjects.

Gait parameters	Total	*n* = 140	Male	*n* = 68	Female	*n* = 72	*p*
	Mean	SD	Mean	SD	Mean	SD	
Total steps	722.86	81.49	720.40	69.20	725.19	92.03	0.727
Walking distance (m)	337.21	133.20	366.03	132.29	310.00	122.99	0.013
Walking speed (km/h)	3.55	1.28	3.86	1.30	3.25	1.20	0.005
Step frequency (cycles/s)	1.00	0.11	1.00	0.09	1.01	0.13	0.780
Step length (cm)	47.96	16.48	52.56	16.91	43.61	14.92	0.001
Vertical COM displacement(cm)	2.13	1.12	2.56	1.25	1.72	0.81	<0.001
Support time (s)	0.67	0.14	0.65	0.13	0.68	0.14	0.234

### 3.2 Correlation analysis of joint angles and gait parameters in males and females

Pearson correlation coefficients were calculated between joint angles at the trunk, hip, knee, and ankle joints and various gait parameters. Significance tests were performed separately for the male and female groups. The present study systematically evaluated the associations between multiple joint angles and gait parameters in male and female subjects through Pearson correlation analysis. The results of the study demonstrated a significant correlation between lower limb joint angles and several parameters of gait, with partial differences observed between male and female subjects.

As demonstrated in Table 3, among male subjects, trunk flexion exhibited a highly significant negative correlation with total step count and step frequency (*r* = −0.435, *p* < 0.001), and a significant positive correlation with stance time (*r* = 0.399, *p* = 0.001). The trunk extension exhibited consistent trends, demonstrating a negative correlation with total step count and step frequency (*r* = −0.456, *p* < 0.001) and a positive correlation with stance time (*r* = 0.419, *p* < 0.001). These correlations are represented in the heatmap by red-orange tones (negative correlation) and blue tones (positive correlation), respectively. Conversely, lateral trunk flexion demonstrated no significant association with any gait parameters (*p* > 0.05).

**Table 3:** Correlation analysis between male joint angles and gait parameters.

Male joint angles and gait parameters	Total steps		Walking distance (m)		Walking speed (km/h)		Step frequency (cycles/s)		Step length (cm)		Vertical COM displacement(cm)		Support Time (s)	
	Pearsons r	*p*-value	Pearsons r	*p*-value	Pearsons r	*p*-value	Pearsons r	*p*-value	Pearsons r	*p*-value	Pearsons r	*p*-value	Pearsons r	*p*-value
Trunk Flexion (°)	−0.435	<0.001	−0.192	0.117	−0.258	0.034	−0.435	<0.001	−0.131	0.286	0.196	0.109	0.399	0.001
Trunk Extension (°)	−0.456	<0.001	−0.228	0.061	−0.301	0.013	−0.458	<0.001	−0.186	0.130	0.103	0.402	0.419	<0.001
Trunk Left Lateral Flexion (°)	−0.059	0.632	−0.163	0.185	−0.198	0.106	−0.053	0.667	−0.179	0.145	0.069	0.575	0.093	0.453
Trunk Right Lateral Flexion (°)	0.066	0.595	0.046	0.707	0.066	0.592	0.056	0.651	0.066	0.595	0.043	0.729	−0.094	0.445
Left Hip Flexion (°)	0.053	0.666	0.534	<0.001	0.538	<0.001	0.073	0.557	0.620	<0.001	0.640	<0.001	−0.096	0.438
Left Hip Extension (°)	0.081	0.511	0.538	<0.001	0.576	<0.001	0.103	0.404	0.671	<0.001	0.575	<0.001	−0.048	0.698
Right Hip Flexion (°)	0.081	0.510	0.557	<0.001	0.553	<0.001	0.100	0.417	0.639	<0.001	0.579	<0.001	−0.094	0.446
Right Hip Extension (°)	0.086	0.487	0.570	<0.001	0.600	<0.001	0.111	0.368	0.685	<0.001	0.601	<0.001	−0.081	0.510
Left Knee Flexion (°)	0.252	0.038	0.643	<0.001	0.645	<0.001	0.266	0.028	0.654	<0.001	0.504	<0.001	−0.352	0.003
Left Knee Extension (°)	−0.177	0.148	−0.072	0.560	−0.181	0.140	−0.172	0.161	−0.192	0.116	0.100	0.417	0.040	0.745
Right Knee Flexion (°)	0.212	0.083	0.619	<0.001	0.610	<0.001	0.232	0.057	0.621	<0.001	0.550	<0.001	−0.274	0.024
Right Knee Extension (°)	−0.135	0.271	−0.211	0.084	−0.299	0.013	−0.146	0.236	−0.302	0.012	−0.101	0.413	0.049	0.694
Left Ankle Dorsiflexion (°)	0.134	0.276	0.579	<0.001	0.600	<0.001	0.155	0.206	0.654	<0.001	0.468	<0.001	−0.149	0.224
Left Ankle Plantarflexion (°)	0.297	0.014	0.573	<0.001	0.641	<0.001	0.308	0.010	0.617	<0.001	0.305	0.011	−0.287	0.018
Right Ankle Dorsiflexion (°)	0.164	0.182	0.594	<0.001	0.609	<0.001	0.190	0.122	0.651	<0.001	0.450	<0.001	−0.171	0.163
Right Ankle Plantarflexion (°)	0.362	0.002	0.530	<0.001	0.593	<0.001	0.364	0.002	0.558	<0.001	0.255	0.036	−0.329	0.006
Left Ankle Pronation (°)	0.086	0.487	0.125	0.311	0.117	0.344	0.100	0.418	0.093	0.452	0.071	0.567	−0.058	0.641
Left Ankle Supination (°)	0.302	0.012	0.536	<0.001	0.606	<0.001	0.331	0.006	0.601	<0.001	0.457	<0.001	−0.322	0.007
Right Ankle Pronation (°)	0.239	0.050	0.197	0.107	0.196	0.110	0.239	0.050	0.134	0.274	0.062	0.617	−0.189	0.123
Right Ankle Supination (°)	0.277	0.022	0.542	<0.001	0.590	<0.001	0.306	0.011	0.610	<0.001	0.503	<0.001	−0.248	0.041

Among core joints, bilateral hip flexion and extension exhibited robust positive correlations with walking distance, walking speed, step length, and vertical COM displacement (*r* = 0.534–0.685, *p* < 0.001). The heatmap illustrates the strong positive associations in dark blue. An examination of the hip angles revealed no substantial correlation with total step count, step frequency, or stance time (*p* > 0.05). This finding is illustrated by the heatmap, which displays the corresponding regions in lighter shades. For knee angles, left knee flexion demonstrated a positive correlation with total steps and step frequency (*r* = 0.252–0.266, *p* < 0.05; *r* = 0.266, *p* = 0.028), and exhibited strong positive correlations with walking distance, walking speed, and step length (*r* = 0.643–0.654, *p* < 0.001). Furthermore, left knee flexion showed a negative correlation with support time (*r* = −0.352, *p* = 0.003). Right knee flexion exhibited analogous trends, albeit with marginally weaker correlations.

A highly significant positive correlation was demonstrated between ankle dorsiflexion, plantarflexion and supination, and walking distance, walking speed and step length (*r* = 0.530–0.654, *p* < 0.001). The application of heatmaps serves to emphasize these strong associations in deep blue, thus indicating their status as key regulators of male gait efficiency. The findings of the study demonstrated a positive correlation between certain angles and total steps and step frequency, while a negative correlation was identified between most angles and support time. These results were consistent with the color transition patterns observed in the heatmap. Furthermore, ankle pronation exhibited no distinct color gradients in the intersection zones with all gait parameters (as shown in Figure 1).

In contrast to the findings observed in males, no statistically significant correlation was identified between female trunk angles, encompassing flexion, extension, and lateral flexion, and any of the measured gait parameters (*p* > 0.05). As demonstrated in Table 4, the corresponding regions in the heatmap predominantly exhibit light tones, devoid of any discernible color gradient.

**Table 4:** Correlation analysis between female joint angles and gait parameters.

Female joint angles and gait parameters	Total steps		Walking distance (m)		Walking speed (km/h)		Step frequency (cycles/s)		Step length (cm)		Vertical COM displacement(cm)		Support time (s)	
	Pearsons r	*p*-value	Pearsons r	*p*-value	Pearsons r	*p*-value	Pearsons r	*p*-value	Pearsons r	*p*-value	Pearsons r	*p*-value	Pearsons r	*p*-value
Trunk Flexion (°)	−0.111	0.355	−0.085	0.476	−0.075	0.530	−0.110	0.358	−0.057	0.635	0.053	0.659	−0.054	0.654
Trunk Extension (°)	−0.114	0.341	−0.106	0.374	−0.100	0.401	−0.113	0.343	−0.091	0.447	−0.031	0.798	−0.071	0.556
Trunk Left Lateral Flexion (°)	0.066	0.580	−0.049	0.682	−0.011	0.929	0.069	0.567	−0.008	0.946	−0.037	0.759	−0.046	0.701
Trunk Right Lateral Flexion (°)	−0.147	0.219	−0.079	0.510	−0.126	0.293	−0.150	0.209	−0.110	0.359	0.045	0.708	0.099	0.406
Left Hip Flexion (°)	0.180	0.130	0.577	<0.001	0.603	<0.001	0.180	0.130	0.667	<0.001	0.658	<0.001	−0.308	0.008
Left Hip Extension (°)	0.191	0.109	0.625	<0.001	0.648	<0.001	0.191	0.108	0.690	<0.001	0.552	<0.001	−0.338	0.004
Right Hip Flexion (°)	0.161	0.175	0.565	<0.001	0.600	<0.001	0.161	0.176	0.678	<0.001	0.624	<0.001	−0.264	0.025
Right Hip Extension (°)	0.224	0.059	0.650	<0.001	0.670	<0.001	0.224	0.058	0.697	<0.001	0.577	<0.001	−0.401	<0.001
Left Knee Flexion (°)	0.268	0.023	0.577	<0.001	0.578	<0.001	0.267	0.023	0.543	<0.001	0.392	0.001	−0.523	<0.001
Left Knee Extension (°)	−0.137	0.251	−0.080	0.505	−0.109	0.360	−0.138	0.249	−0.097	0.419	−0.008	0.947	0.162	0.174
Right Knee Flexion (°)	0.316	0.007	0.581	<0.001	0.588	<0.001	0.315	0.007	0.547	<0.001	0.330	0.005	−0.561	<0.001
Right Knee Extension (°)	−0.244	0.039	−0.240	0.042	−0.270	0.022	−0.246	0.037	−0.238	0.044	−0.069	0.567	0.318	0.006
Left Ankle Dorsiflexion (°)	0.187	0.116	0.574	<0.001	0.594	<0.001	0.191	0.108	0.634	<0.001	0.471	<0.001	−0.387	0.001
Left Ankle Plantarflexion (°)	0.274	0.020	0.559	<0.001	0.566	<0.001	0.273	0.020	0.592	<0.001	0.363	0.002	−0.495	<0.001
Right Ankle Dorsiflexion (°)	0.167	0.161	0.653	<0.001	0.647	<0.001	0.172	0.149	0.668	<0.001	0.558	<0.001	−0.393	0.001
Right Ankle Plantarflexion (°)	0.304	0.009	0.541	<0.001	0.551	<0.001	0.304	0.009	0.570	<0.001	0.303	0.010	−0.489	<0.001
Left Ankle Pronation (°)	0.106	0.375	0.078	0.513	0.112	0.351	0.107	0.372	0.083	0.489	0.056	0.639	−0.282	0.016
Left Ankle Supination (°)	0.382	0.001	0.518	<0.001	0.520	<0.001	0.379	0.001	0.489	<0.001	0.467	<0.001	−0.429	<0.001
Right Ankle Pronation (°)	0.111	0.353	0.200	0.092	0.226	0.057	0.111	0.352	0.177	0.137	0.079	0.507	−0.310	0.008
Right Ankle Supination (°)	0.434	<0.001	0.521	<0.001	0.532	<0.001	0.432	<0.001	0.478	<0.001	0.445	<0.001	−0.498	<0.001

Bilateral hip flexion and extension showed positive correlations with walking distance, walking speed, step length, and vertical COM displacement (*r* = 0.567–0.697, *p* < 0.001). The intensity of deep blue hues in the heatmap was greater than in the male heatmap, indicating stronger association strength. Bilateral knee flexion showed positive correlations with total steps and step frequency (*r* = 0.267–0.316, *p* < 0.05), and strong positive correlations with walking distance, walking speed, and step length (*r* = 0.543–0.588, *p* < 0.001), and showed a strong negative correlation with support time (*r* = −0.523–0.561, *p* < 0.001). The color saturation in the heatmap was higher than that in the male heatmap. Notably, right knee extension showed a negative correlation with walking distance, walking speed, and step length (*r* = −0.238 to −0.270, *p* < 0.05), with the corresponding area in the heatmap appearing in light red tones. This association was not observed in males.

Additionally, ankle dorsiflexion, plantarflexion, and supination exhibited strong positive correlations with walking distance, walking speed, and step length (*r* = 0.518–0.688, *p* < 0.001), as illustrated by the deep blue hues in the heatmap. The negative correlation with support time was more pronounced (*r* = −0.282 to −0.498, *p* < 0.005), as demonstrated by the more vivid color contrasts in the heatmap. Furthermore, partial correlations were observed for ankle pronation in females: left ankle pronation negatively correlated with support time (*r* = −0.282, *p* = 0.016), while right ankle pronation approached statistical significance with walking speed (*r* = 0.226, *p* = 0.057), with subtle color changes in the corresponding heatmap regions. This pattern was not observed in males (as shown in Figure 2).

### 3.3 Regression analysis of joint angles and gait parameters in males and females

Regression analysis was conducted on all parameters reaching statistical significance in the correlation analysis (Tables 5, 6). The following results were obtained: For males, all models achieved statistical significance. The researchers explained variations in gait parameters within an R^2^ range of 26.7–79.51% and an adjusted *R*^2^ range of 16.77–74.58%. For the female subjects, all models also attained statistical significance, with R^2^ ranging from 24.13–79.89% and adjusted *R*^2^ from 15.84–75.38%. The explanatory power of the models varied based on the number of independent variables (joint angles) and their predictive capability.

**Table 5:** Regression analysis between male independent joint angles and gait parameters.

Male independent joint angles and gait parameters	Total steps	Walking distance (m)	Walking speed (km/h)	Step frequency (cycles/s)	Step length (cm)	Vertical COM displacement(cm)	Support time (s)
joint angles and	×		×	×			×
gait parameters	×		×	×			×
Trunk Left Lateral Flexion (°)							
Trunk Right Lateral Flexion (°)							
Left Hip Flexion (°)		×	×		×	×	
Left Hip Extension (°)		×	×		×	×	
Right Hip Flexion (°)		×	×		×	×	
Right Hip Extension (°)		×	×		×	×	
Left Knee Flexion (°)	×	×	×	×	×	×	×
Left Knee Extension (°)							
Right Knee Flexion (°)		×	×		×	×	×
Right Knee Extension (°)			×		×		
Left Ankle Dorsiflexion (°)		×	×		×	×	
Left Ankle Plantarflexion (°)	×	×	×	×	×	×	×
Right Ankle Dorsiflexion (°)		×	×		×	×	
Right Ankle Plantarflexion (°)	×	×	×	×	×	×	×
Left Ankle Inversion (°)							
Left Ankle Eversion (°)	×	×	×	×	×	×	×
Right Ankle Inversion (°)	×						
Right Ankle Eversion (°)	×	×	×	×	×	×	×
*R* ^2^	0.267	0.6348	0.7312	0.2767	0.7951	0.6598	0.3821
Adjusted *R*^2^	0.1677	0.5551	0.6536	0.1923	0.7458	0.5855	0.2984
*p*-value	0.01371	2.336e-08	4.193e-10	0.005	3.695e-14	3.989e-09	0.0002316

Regression analysis between independent joint angles (predictor variables) and gait parameters (dependent variables). “X” marks significant joint angles.

**Table 6:** Regression analysis between female independent joint angles and gait parameters.

Female independent joint angles and gait parameters	Total steps	Walking distance (m)	Walking speed (km/h)	Step frequency (cycles/s)	Step length (cm)	Vertical COM displacement(cm)	Support time (s)
Trunk Flexion (°)							
Trunk Extension (°)							
Trunk Left Lateral Flexion (°)							
Trunk Right Lateral Flexion (°)							
Left Hip Flexion (°)		×	×		×	×	×
Left Hip Extension (°)		×	×		×	×	×
Right Hip Flexion (°)		×	×		×	×	×
Right Hip Extension (°)		×	×		×	×	×
Left Knee Flexion (°)	×	×	×	×	×	×	×
Left Knee Extension (°)							
Right Knee Flexion (°)	×	×	×	×	×	×	×
Right Knee Extension (°)	×	×	×	×	×		×
Left Ankle Dorsiflexion (°)		×	×		×	×	×
Left Ankle Plantarflexion (°)	×	×	×	×	×	×	×
Right Ankle Dorsiflexion (°)		×	×		×	×	×
Right Ankle Plantarflexion (°)	×	×	×	×	×	×	×
Left Ankle Inversion (°)							×
Left Ankle Eversion (°)	×	×	×	×	×	×	×
Right Ankle Inversion (°)							×
Right Ankle Eversion (°)	×	×	×	×	×	×	×
*R* ^2^	0.2414	0.7331	0.7489	0.2413	0.7989	0.5879	0.3874
Adjusted *R*^2^	0.1584	0.6732	0.6926	0.1583	0.7538	0.5041	0.2233
*p*-value	0.0105	3.08e-12	5.859e-13	0.0105	1.313e-15	1.043e-07	0.01048

Regression analysis between independent joint angles (predictor variables) and gait parameters (dependent variables). “X” marks significant joint angles.

## 4 Discussion

This study systematically revealed gender differences and associations between trunk, hip, knee, and ankle joint range of motion and gait parameters in older adults, based on treadmill gait data. It should be noted that anthropometric variables such as height, weight, and leg length were not included in the regression models. The core reason is that the study focuses on “gender-specific associations between joint angles and gait parameters” rather than quantifying the independent effects of anthropometric variables on gait. Additionally, the gait parameters and joint angles used in this study are all relative indicators, whose inherent properties have reduced the confounding interference caused by absolute size. This design is consistent with the conventions of similar gait studies focusing on gender differences (7, 8). Notably, the age-related decline in walking speed reported to decrease by 12–16% per decade was mitigated by the balanced age distribution across gender groups (males: 69.2 ± 6.8 years; females: 69.9 ± 6.0 years, *p* = 0.679). This symmetry ensures that age-related gait changes affected both genders equally, without interfering with the identification of gender-specific associations—our core research focus. Consistent with prior literature (8), gender-specific gait biomechanics are primarily driven by physiological differences (e.g., pelvic structure, muscle strength distribution) rather than age, further supporting the robustness of our findings. It should be noted that treadmill walking differs from overground walking in terms of environmental complexity (e.g., terrain variability, obstacle avoidance) and sensory feedback (15), which may limit the generalizability of the findings to real-life overground walking. However, the Walker View treadmill’s SCX adaptive speed control system can real-time track the user’s COM and automatically adjust the belt speed, simulating the natural rhythm of overground walking and reducing the speed difference between traditional treadmills and overground walking. Lippi (14) further confirmed that this adaptive speed control feature enhances the ecological validity of treadmill-based gait assessment in obese post-ICU patients. Providing a reliable basis for the subsequent analysis of gender-specific gait characteristics.

Building on the aforementioned methodology, our analysis identified distinct gender differences in both joint angles and their associations with gait parameters. Firstly, it was determined that male subjects within this demographic exhibited greater angles of hip extension and ankle plantar flexion, while female subjects demonstrated greater angles of knee extension and ankle pronation. This finding is consistent with the report by Mullerpatan et al. (16), which demonstrated that male subjects exhibited greater hip adduction and pelvic anterior tilt angles compared to female subjects. In the present study, female subjects exhibited greater knee extension angles, that is to say, smaller knee flexion angles. This finding is consistent with the results reported by Araki (8), who observed a faster decline in knee flexion angles in women. This suggests that knee joint mobility deteriorates earlier in females. With regard to spinal and trunk angles, males exhibited greater forward bending angles, while females demonstrated larger angles related to trunk lateral flexion and rotation compared to males. This finding suggests that senior females rely more on trunk stability during gait to maintain walking efficiency. The aforementioned gender differences may be attributable to variations in pelvic structure and hip and knee muscle strength (17).

In gait parameters, differences between males and females align with most reports: males of the same age typically exhibit greater step length and higher walking speed, while females maintain gait through higher step frequency (18). Our data also show that males have overall greater step length and walking speed than females (*p* < 0.05), while females have slightly longer double-support time, reflecting a potentially more conservative walking pattern. These findings align with prior studies on older adults’ gait. For instance, Inns et al. noted that older adults exhibit reduced walking speed, slower step frequency, and increased double support time compared to younger individuals (19). Additionally, Frimenko’s review (7) indicated that gender differences in walking speed largely stem from height variations, without affecting the fundamental gender differences in step length and step frequency.

Subsequent correlation and regression analyses revealed that different joint angles contribute variably to walking function. A salient finding of this study is that, in males, trunk angle exhibits a significant correlation with step count, step frequency, and support time (*p* < 0.001). Conversely, no such association is observed in females (*p* > 0.05). This discrepancy may be attributable to the differential gait compensation mechanisms observed between genders. With the progression of age, male trunk muscle groups undergo a more gradual decline in comparison to lower limb muscles. Consequently, males tend to maintain balance during walking by adjusting trunk angle. Moderate trunk extension has been shown to enhance stability during the stance phase (20), thereby reducing the risk of falling – a finding that is consistent with this study’s observation of a positive correlation between male trunk extension and stance duration. Conversely, women experience accelerated torso muscle degeneration, with muscle loss rates 1.2 times higher per decade than in men (21). This diminished torso regulation capacity results in no significant correlation between torso angle and gait parameters, leading women to rely instead on lower limb joint flexibility to maintain gait. Across all participants, the most significant correlations were observed between bilateral hip flexion/extension and knee flexion angles, and walking distance, walking speed, step length, and support time. This finding suggests that hip flexion/extension capacity and knee flexion mobility are pivotal factors in preserving increased step length and expedited walking speed. This finding is consistent with the findings of previous research in this field. For instance, Chung used gait analysis to demonstrate a close relationship between hip extension and ankle plantar flexion strength and walking speed (22). Similarly, Eliane (23) and Eduardo (24) emphasized that reduced ankle joint mobility significantly decreases walking speed.

We also found that dorsiflexion and plantar flexion angles at the ankle joint significantly influence walking speed and step length. This aligns with the findings of Pol’s systematic review (25), which reported that plantarflexion angles in older individuals are significantly lower than in younger adults, thereby limiting the generation of propulsive force. In regression models, ankle plantarflexion angle repeatedly entered both stride length and speed models, further supporting the notion that enhancing ankle joint flexibility may improve walking ability in the senior citizens. Additionally, this study found a negative correlation between right knee extension and walking speed in females (*r* = −0.270, *p* = 0.022), a phenomenon not observed in males. A possible explanation is that excessive knee extension in females may lead to premature knee locking during the late stance phase, reducing the time available for ankle plantar flexion force generation and consequently decreasing propulsive efficiency (26). This suggests an optimal range for knee extension in women, where excessive extension may actually impair gait, providing a precise target for clinical intervention.

This study identified gender-specific associations between ankle pronation angle and gait parameters in women: left ankle pronation negatively correlated with support time (*r* = −0.282,*p* = 0.016), while right ankle pronation showed a near-positive correlation with walking speed (*r* = 0.226, *p* = 0.057). In men, ankle pronation exhibited no significant correlations with any gait parameters (*p* = 0.016), while right ankle pronation showed a near-positive correlation with walking speed (*r* = 0.226, *p* = 0.057). In contrast, ankle pronation in males showed no significant association with any gait parameters (*p* > 0.05). From a gait biomechanics perspective, ankle pronation is a composite movement involving dorsiflexion and adduction (27). Its core function is to cushion ground impact forces during the stance phase, adjust arch loading to enhance foot-ground adaptation, and thereby shorten support time by optimizing balance regulation efficiency. This reduces ground adaptation time, indirectly creating conditions for increased walking speed. Combined with the larger knee extension angles in females and greater ankle plantar flexion angles in males observed in this study, this suggests relatively weaker lower limb propulsive muscle strength in females (28). This association may represent a gender-specific compensatory balance strategy: when overall lower-limb propulsive force is insufficient, women optimize ground adaptation and balance maintenance efficiency during the stance phase by finely regulating the amplitude and timing of ankle pronation (29), thereby preventing gait sluggishness and maintaining stable walking speed. Men, benefiting from superior propulsive function in larger joints like hip extension and ankle plantar flexion, maintain adequate gait without relying on precise ankle pronation adjustments. This finding refines the biomechanical characteristics of female gait regulation. Compared to males who rely on large-joint propulsive forces, females depend more on balance regulation through complex ankle movements (30). This provides a precise target for ankle pronation muscle coordination training in gait interventions tailored for women and fills a gap in understanding the gait regulation mechanisms of healthy senior females.

However, this study has several limitations. The cross-sectional design of the study precludes establishing a causal relationship between changes in joint range of motion over time and gait deterioration, and all interpretations are based on associations rather than causal inferences. The sample size was limited and exclusively included healthy, community-dwelling independent older adults, excluding those with significant gait impairment or neurological disorders. Consequently, the findings may not be applicable to all older adult populations. Notably, we did not assess the Foot Posture Index (FPI), which may influence gait parameters and joint angles; this should be considered in future studies to ensure sample homogeneity. Further, physical activity level was evaluated via a brief questionnaire rather than objective measurements, which may introduce residual confounding. Additionally, gait was measured on a treadmill or in a near-static environment, neglecting the impact of complex walking conditions. Future studies should validate these findings in more everyday settings. Finally, this study analyzed simplified indicators like sagittal plane joint motion without examining detailed differences across the full kinematic cycle. Given that gait is a whole-body coordinated movement, trunk and pelvic motion, along with joint movements in other planes, may also influence gait parameters. Future research integrating additional kinematic features and dynamic parameters could enhance our understanding of gait mechanisms.

## 5 Conclusion

This study, which is based on data from healthy older adults, indicates significant gender differences in trunk, hip, knee, and ankle joint angles, as well as gait parameters. It has been demonstrated that males demonstrate greater hip extension, ankle plantar flexion, and supination angles. Concurrently, males exhibit superior walking speed and step length. It has been demonstrated that females exhibit greater knee extension angles and greater reliance on lower limb joint flexibility during gait. The association between joint angles and gait parameters exhibits gender specificity: male trunk flexion/extension angles contribute to gait regulation, whereas no such association exists in females. The findings of this study demonstrate a robust positive correlation between hip, knee, and ankle angles, irrespective of gender, and walking speed and step length. However, it is noteworthy that female subjects exhibit a more pronounced correlation. Lower limb joint angles have been shown to be significant predictors of gait parameters in older adults, accounting for 24.14 to 79.89% of gait variability. The study recommends that gender differences be fully considered in gait function assessment and rehabilitation training for older adults, with targeted improvements in lower limb joint angles to promote safer and more efficient walking ability.

## Data Availability

The raw data supporting the conclusions of this article will be made available by the authors, without undue reservation.
